# Rethinking Otorhinolaryngologic Care Pathways in Children with Down Syndrome: A Multidisciplinary Framework for Early Diagnosis and Management

**DOI:** 10.3390/jcm14113889

**Published:** 2025-06-01

**Authors:** Francesco Fabrizio Comisi, Elena Esposito, Salvatore Savasta

**Affiliations:** Pediatric Clinic and Rare Diseases, Microcitemico Hospital “A.Cao”, Department of Medical Sciences and Public Health, University of Cagliari, 09121 Cagliari, Italy

**Keywords:** down syndrome, hearing loss, otitis media, obstructive sleep apnea, multidisciplinary care, pediatric ENT

## Abstract

**Background:** Children with Down syndrome (DS) are at high risk for a broad spectrum of otorhinolaryngologic (ENT) disorders, including hearing impairment, obstructive sleep apnea (OSA), dysphagia, and language delay. These conditions often coexist and interact with the neurodevelopmental and anatomical features of DS, requiring early identification and coordinated management. Despite the clinical burden, ENT involvement in DS remains under-characterized and inconsistently addressed in care pathways. **Methods:** A narrative review was conducted to provide an integrative overview of ENT manifestations in pediatric patients with DS. A literature search was performed in PubMed, Scopus, and Web of Science, covering studies published between 1979 and 2025. Articles were included if they addressed ENT disorders in children with DS and met clinical relevance criteria. A total of 45 studies were selected and analysed by study design, focus, and contribution to diagnostic or therapeutic frameworks. **Results:** The majority of included studies were observational in nature, covering key domains such as conductive hearing loss, chronic otitis media with effusion, sleep-disordered breathing, and feeding/swallowing dysfunction. Several articles emphasized the importance of early audiologic and polysomnographic screening. Gaps in standardization and accessibility of multidisciplinary ENT care were consistently reported. A proposed framework for integrated evaluation is discussed. **Conclusions:** ENT manifestations in pediatric DS are frequent, multifactorial, and clinically impactful. A multidisciplinary, anticipatory model of care is essential for timely diagnosis and targeted intervention. This review highlights the need to formalize ENT pathways within comprehensive care protocols for children with DS.

## 1. Introduction

Down syndrome is the most common chromosomal disorder among live-born infants, occurring in approximately 1 in every 700 to 800 births. It is caused by the presence of an extra copy of chromosome 21, typically due to nondisjunction during maternal meiosis [1]. DS presents with a broad phenotypic spectrum, including intellectual disability, distinctive craniofacial features, and a high prevalence of congenital anomalies involving the cardiac, gastrointestinal, and skeletal systems. Diagnosis is confirmed through cytogenetic analysis, employing techniques such as karyotyping or fluorescence in situ hybridization (FISH) [2]. Developmental delay and cognitive impairment are key features, though their severity may vary. A subset of individuals exhibit mosaicism, a condition characterized by the coexistence of trisomic and euploid cell lines, which is often associated with a milder clinical phenotype. Regardless of genetic subtype, individuals with DS frequently face a wide set of medical challenges, among which otolaryngological complications are particularly prominent [3,4]. ENT manifestations in DS include recurrent acute otitis media (AOM), both sensorineural and conductive hearing loss, speech and language delays, and an increased risk of sleep-disordered breathing. These manifestations are often attributable to underlying craniofacial and neuromuscular anomalies. Given the high prevalence and multifactorial nature of ENT-related conditions in patients with Down syndrome, early and comprehensive otolaryngologic assessment is essential to achieve optimal clinical outcomes [5]. To address these challenges, this narrative review aims to synthesize current evidence on otolaryngological manifestations in children with Down syndrome, highlighting diagnostic challenges, therapeutic strategies, and the role of multidisciplinary care.

## 2. Materials and Methods

This narrative review was conducted to synthesize current knowledge on otolaryngological manifestations in pediatric patients with Down syndrome, focusing on conditions such as hearing impairment, obstructive sleep apnea, language delay, dysphagia, and upper airway abnormalities. The objective was to provide a clinically relevant overview by integrating evidence from both historical and contemporary sources. A comprehensive literature search was performed using databases including PubMed, Scopus, and Web of Science, covering publications from May 1979 to March 2025. Search terms included combinations of “Down syndrome”, “Trisomy 21”, “pediatric”, “hearing loss”, “obstructive sleep apnea”, “ENT disorders”, “Otolaryngologic manifestations”, “dysphagia”, “language development”, and “feeding difficulties”. Additional references were identified through manual screening of the bibliographies of selected articles. Inclusion criteria encompassed original research articles (observational or interventional studies), clinical guidelines, expert consensus documents, systematic and narrative reviews, and case series that reported on ENT-related conditions in children with DS (aged < 18 years). Studies focusing exclusively on adults or not addressing ENT manifestations were excluded unless pediatric data were explicitly reported. A total of 45 articles met the inclusion criteria. The majority were observational studies, while others comprised systematic or narrative reviews, clinical practice guidelines, and case-based reports. The inclusion of older studies, some published before 2000, was justified by their historical relevance, foundational observations, or continued clinical applicability. This broader temporal scope allowed for a longitudinal perspective on evolving diagnostic and therapeutic approaches. Given the narrative nature of this review and the heterogeneity of sources, no formal risk of bias assessment was performed. The synthesis aimed to integrate findings across a wide methodological spectrum, offering a contextually rich and clinically oriented perspective on ENT involvement in children with Down syndrome.

## 3. ENT Manifestations

Children with DS exhibit distinctive craniofacial dysmorphic features that affect essential functions such as feeding, respiration, and speech. The typical phenotype includes brachycephaly, midfacial and mandibular hypoplasia, macroglossia, a flat nasal bridge, and underdeveloped paranasal sinuses—traits linked to increased developmental instability associated with trisomy 21 [6]. These anatomical characteristics contribute to widespread dysfunction of the upper aerodigestive tract. A high incidence of lower airway anomalies has been reported in children with DS, particularly airway malacia, including laryngomalacia and tracheomalacia, as well as, less frequently, tracheal bronchus or subglottic stenosis. These forms of malacia may result in dynamic airway collapse during inspiration or sleep and should be carefully assessed in symptomatic patients [7]. Structural anomalies also lead to oropharyngeal dysfunction, with impaired orosensorimotor coordination and a high prevalence of dysphagia across the oral, pharyngeal, and esophageal phases [4]. A UK National Health Service (NHS) evaluation reported that over 50% of children with DS showed clinical signs of dysphagia [8]. Among those who underwent instrumental swallowing assessments, 90.2% exhibited silent aspiration, characterized by the absence of overt symptoms such as coughing or wheezing. Early diagnosis and management before 12 months of age were associated with fewer respiratory complications, whereas delayed or absent intervention correlated with increased lower respiratory tract infections (LRTIs) [8]. Contributing factors include chewing inefficiency, poor bolus control, tongue thrust, and a delayed swallowing reflex [4,9]. The same craniofacial traits—macroglossia, hypotonia, and pharyngeal narrowing—also predispose to upper respiratory tract infections (URTIs), airway obstruction, chronic nasal discharge, and sinusitis, thereby increasing the risk of obstructive sleep apnea (OSA) [4,10]. Craniofacial hypoplasia and narrow nasal cavities further exacerbate nasal obstruction, often worsened by adenoidal hypertrophy and enlarged turbinates. Recurrent or persistent rhinitis and sinusitis are common and may compromise nasal breathing and sleep quality [11]. Speech development is likewise affected, with contributing factors including reduced oral cavity volume, impaired articulatory precision, and delayed or atypical phonological development—all of which significantly reduce speech intelligibility [12,13]. Together with OSA and dysphagia, these findings reinforce the need for early, multidisciplinary ENT-focused interventions to support development in this population. Such strategies play a critical role in improving quality of life and developmental outcomes in children with DS [14,15].

### 3.1. Hearing Impairment

**Figure 1:**
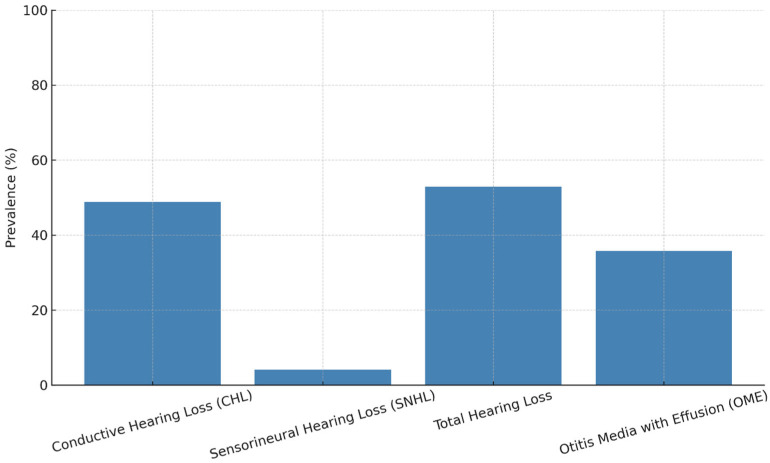
Prevalence of hearing loss and otitis media with effusion in children with Down syndrome. Data derived from [16].

Children with Down syndrome are at increased risk for hearing loss, which can deeply impact their speech and language development [14]. The types of hearing impairment observed in this population include conductive, sensorineural, and mixed hearing loss. Conductive hearing loss is the most prevalent during early childhood and is frequently associated with chronic otitis media with effusion (OME) [1]. This condition is due to anatomical and functional abnormalities of the eustachian tube, heightened susceptibility to infections, and the craniofacial dysmorphisms commonly observed in DS. Prevalence rates of OME in this population are notably high, with studies reporting up to 93% of children affected by age 1, and 68% still affected at age 5 [14]. Although less frequent, sensorineural hearing loss, resulting from cochlear or auditory nerve dysfunction, can also occur, either independently or in conjunction with conductive loss, leading to mixed hearing impairment [14]. The presence of hearing loss during critical periods of language acquisition, particularly between ages 2 and 4, has been shown to significantly impair the development of both expressive and receptive language abilities in children with DS [16]. A recent cross-sectional study conducted in Egypt on 170 pediatric patients found that 52.9% of children with DS had hearing loss, predominantly conductive (48.8%), with OME identified in 35.8% of cases [16] (Figure 1). These findings underscore the importance of systematic audiological assessment in this population. Children with moderate to severe hearing impairment during early developmental stages score lower on standardized assessments of vocabulary, language comprehension, and speech production when compared to their peers with normal hearing or only mild, fluctuating hearing loss [14]. Notably, these language deficits persist even after controlling for chronological age and nonverbal cognitive ability, suggesting that hearing loss exerts an independent and additive effect on linguistic outcomes [17]. Furthermore, speech in children with DS is frequently characterized by reduced intelligibility, impaired phonological accuracy, and delays or atypical patterns in the acquisition of syntax and morphology [18]. Contributing factors include anatomical features such as macroglossia and generalized hypotonia, which interfere with articulatory precision, while fluctuating hearing thresholds compromise auditory feedback critical for phonological development [12]. Early identification and regular audiological monitoring are essential. Integration of audiological and speech-language therapeutic interventions is strongly recommended to prevent or mitigate long-term communicative deficits [19].

### 3.2. Upper Respiratory Tract Infections

Children with Down syndrome show a highly increased incidence of recurrent otitis media, with studies reporting hearing loss in up to 77% of cases, most commonly resulting from persistent middle ear effusion and eustachian tube dysfunction [20,21,22]. This elevated prevalence is attributed to a constellation of anatomical and physiological risk factors, including craniofacial dysmorphisms, stenotic external auditory canals, and immunologic abnormalities such as ciliary dyskinesia [8,12,23]. These factors contribute to chronic OME, which, if left untreated, may adversely affect language acquisition and cognitive development [3].

As a consequence of the anatomical abnormalities and immature immunologic development frequently seen in children with Down syndrome, chronic nasal drainage and sinusitis are common clinical findings [4].

### 3.3. Obstructive Sleep Apnea

Obstructive sleep apnea is highly prevalent among individuals with Down syndrome, affecting up to 76% of children and approaching universal prevalence in adulthood [5,24]. This elevated risk is largely attributable to hallmark anatomical and physiological traits such as generalized hypotonia, macroglossia, and midfacial hypoplasia, which together increase upper airway collapsibility during sleep [25]. In a subset of patients, central sleep apnea may also occur. Although its pathophysiology remains unclear, a defective hypoxic ventilatory drive, similar to that seen in congenital central hypoventilation syndrome (Ondine’s curse), has been proposed [26]. If left untreated, OSA can result in severe complications, such as pulmonary hypertension and progressive cognitive decline [14].

### 3.4. Dysphagia

Dysphagia is a common and clinically significant issue in individuals with Down syndrome (DS), arising from a combination of anatomical and functional abnormalities affecting the upper aerodigestive tract [27]. Hallmark features such as hypotonia, macroglossia, and craniofacial dysmorphisms impair coordination of the oral and pharyngeal phases of swallowing [28]. High-resolution manometry studies have identified reduced pharyngeal contractility and impaired upper esophageal sphincter (UES) relaxation, indicating neuromuscular dysfunctions that compromise safe and efficient swallowing [29]. Children and adolescents with DS often exhibit delayed oral motor development, impaired tongue control, and sensory processing deficits, further exacerbating feeding difficulties and increasing the risk of aspiration, recurrent respiratory infections, and suboptimal nutritional status [30]. Nutritional studies confirm that these impairments can contribute to growth faltering, underscoring the importance of early, targeted feeding interventions [30].

## 4. Diagnosing and Monitoring

In addition to the well-known anatomic peculiarities associated with Down syndrome, airway malacia—including laryngomalacia and tracheomalacia—and, less frequently, the presence of a tracheal bronchus or subglottic stenosis, either in isolation or in combination, are relatively common, particularly in patients with chronic respiratory symptoms. Therefore, endoscopic evaluation and imaging studies may be warranted, especially when respiratory symptoms are recurrent, to better delineate the underlying problems [7].

### 4.1. Hearing Impairment

Down syndrome predisposes to a highly increased risk of hearing loss, with prevalence estimates ranging from 38% to 78%, compared to approximately 2.5% in the general pediatric population [20,21]. Diagnostic accuracy is often limited by anatomical factors, including stenotic external auditory canals and frequent cerumen impaction, which limit the accuracy of direct otoscopic visualization and complicate conventional audiologic assessments [21]. The introduction of universal newborn hearing screening (UNHS), particularly through automated auditory brainstem response (AABR), has significantly enhanced early detection [17]. However, even infants who pass initial screening may subsequently develop conductive or sensorineural hearing loss, underscoring the importance of ongoing surveillance throughout early childhood. Current clinical guidelines recommend an initial audiological evaluation by 3 months of age, followed by periodic assessments. Nonetheless, studies indicate that audiologic testing is most reliable either before 6 months or after 10 months of age, as high rates of inconclusive results are frequently reported within this intermediate window [21]. Objective methods such as auditory brainstem response (ABR) and otoacoustic emissions (OAE) are preferred for infants under 9 months, whereas behavioural techniques, like visual reinforcement audiometry, become feasible and more accurate beyond this age. Continued follow-up is essential due to the fluctuating and, in some cases, progressive nature of hearing impairment in DS, with a considerable proportion of children requiring long-term audiological monitoring and intervention [31]. In cases of profound sensorineural hearing loss, cochlear implantation may be considered [23]. However, the decision to proceed with surgery and the postoperative outcomes are influenced by a range of cognitive and anatomical comorbidities commonly associated with DS. Ultimately, timely diagnosis combined with individualized, developmentally appropriate follow-up strategies is critical for optimizing language acquisition and improving quality of life in this high-risk population [18].

### 4.2. Upper Respiratory Tract Infections

Recurrent ear diseases in children with Down syndrome must be promptly diagnosed and monitored starting shortly after birth, with coordinated care from both the pediatrician and the otolaryngologist. Meticulous ear cleaning, regular examinations, laboratory testing when needed and timely medical and surgical treatments are paramount [23].

A thorough work-up for chronic rhinorrhea and sinusitis starts with ruling out mechanical obstruction of the upper airway. Lateral neck radiographs and/or flexible nasopharyngoscopy can reveal whether enlarged adenoids are blocking the posterior choanae. In protracted or refractory cases, an immunologic evaluation is advisable, including quantitative IgG (with subclass analysis), IgA, IgM, IgE, specific antibody titers to diphtheria, tetanus and pneumococcus, and, when appropriate, complement screening. Allergy testing remains prudent—especially when there is a family history of atopy. Finally, modifiable risk factors such as exposure to cigarette smoke should be identified and eliminated [4].

### 4.3. Obstructive Sleep Apnea

Polysomnography (PSG) is the gold standard for diagnosing OSA and is recommended even in asymptomatic children with DS, given the high prevalence of subclinical disease [5,24,32].

### 4.4. Dysphagia

Otolaryngologists play a key role in the diagnostic pathway, particularly through endoscopic and manometric evaluations [29]. A comprehensive and individualized assessment—integrating ENT examination, functional testing, and nutritional evaluation—is essential to guide therapy. Given the chronic and evolving nature of dysphagia in DS, longitudinal monitoring allows adaptation of care plans across developmental stages [28].

## 5. Management

The management of children with Down syndrome requires a comprehensive multidisciplinary approach due to the wide spectrum of medical, developmental, and functional challenges associated with the condition [3]. Key members of the care team include the pediatrician, otolaryngologist, audiologist, speech-language pathologist, neuropsychomotor therapist, nutritionist, gastroenterologist, phoniatrician, and psychologist, among others. Such collaboration is essential to address complex and interrelated issues, particularly feeding and swallowing difficulties arising from hypotonia, craniofacial dysmorphisms, and impaired orosensorimotor control. Nutritional support is crucial, as children with DS frequently experience growth challenges, including both failure to thrive and obesity, due to altered metabolic regulation and gastrointestinal dysfunction [2,20,33]. Simultaneously, hearing loss requires early audiologic assessment and longitudinal monitoring. Anatomical abnormalities may often require a specific—sometimes surgical—approach. Each case must be carefully evaluated by specialists and treated as needed [7].

### 5.1. Management of Hearing Loss

Management strategies for conductive hearing loss include hearing aids, which have demonstrated favourable outcomes. Transtympanic ventilation tubes (grommets) are selectively employed, especially in patients with otalgia or recurrent suppurative otitis media [23,34]. Table 1 presents a comparative overview of hearing aids and tympanostomy tubes, detailing their respective indications, associated risks, and parental considerations in the treatment of conductive hearing loss in children with DS. Although grommet insertion may yield hearing threshold improvements comparable to those achieved with hearing aids, it is associated with higher procedural risks due to frequent comorbidities and the complex otologic anatomy characteristic of this population [35]. Moreover, qualitative parental feedback suggests that hearing aids are associated with lower levels of emotional and physical distress when compared to surgical intervention, indicating that grommet insertion should be reserved for specific clinical indications rather than employed as a routine management strategy [31,35,36,37].

Interventions should be coordinated by otolaryngologists and audiologists, in close collaboration with speech-language pathologists to ensure appropriate auditory rehabilitation and speech development [18]. Expressive language impairments, often compounded by hearing deficits and generalized hypotonia, demand personalized speech-language therapy. In selected cases, augmentative and alternative communication (AAC) systems may be necessary to support functional communication [31,38]. The inclusion of neuropsychomotor therapists plays an important role in fostering gross and fine motor skills, while recent advances in digital and virtual rehabilitation technologies have shown promising outcomes in enhancing visuomotor integration and cognitive development.

### 5.2. Management of Upper Respiratory Tract Infections

Children with Down syndrome frequently experience recurrent ear diseases, particularly conductive hearing loss due to Eustachian tube dysfunction and chronic otitis media. Management strategies include watchful waiting, hearing aids, and grommet insertion. Studies show that hearing aids are highly effective and better tolerated, offering significant improvement in quality of life with minimal distress compared to surgical interventions [12,20].

In individuals with Down syndrome, the medical treatment of chronic rhinorrhea and sinusitis generally follows the same principles used for the broader population—namely, targeted antibiotics supplemented by intranasal corticosteroids, antihistamines, and decongestants. Because their smaller nasal passages predispose to frequent crusting, regular irrigation with isotonic saline helps cleanse and hydrate the mucosa. Intranasal steroid sprays are effective for mild allergic or vasomotor rhinitis, while second-generation antihistamines such as loratadine, fexofenadine, or cetirizine may be added for further relief.

### 5.3. Management of OSAS

First-line treatment typically involves adenotonsillectomy (AT); however, its efficacy in this population is limited, with symptom resolution achieved in only 6–33% of cases—substantially lower than in typically developing peers. Consequently, many patients require adjunctive therapies. Continuous positive airway pressure (CPAP) is the most commonly used second-line treatment and has demonstrated improvements in apnea–hypopnea index (AHI), behavior, and sleep quality [39]. Despite its effectiveness, CPAP adherence remains a significant barrier due to anatomical challenges (e.g., craniofacial configuration, macroglossia) and neurocognitive factors such as intellectual disability and behavioral dysregulation [40]. Drug-induced sleep endoscopy (DISE) offers valuable insight into the dynamic nature of upper airway obstruction in this group. By simulating natural sleep conditions pharmacologically, DISE allows for accurate identification of obstruction sites and guides tailored surgical or non-surgical interventions [41]. Hypoglossal nerve stimulation (HNS) has recently emerged as a promising alternative for patients who are CPAP-intolerant. By synchronizing nerve stimulation with inspiration, HNS improves airway patency and directly addresses the pathophysiology of OSA. Studies in pediatric and young adult populations with DS have demonstrated significant reductions in AHI and improved quality of life, with good tolerability [42,43]. Given the multifactorial nature of sleep-disordered breathing in DS, a multidisciplinary approach is essential. This should include behavioral desensitization programs, individualized mask fitting, caregiver support, and close follow-up to enhance treatment adherence and long-term outcomes [32,44].

### 5.4. Management of Dysphagia

Effective management of dysphagia requires coordinated input from otolaryngologists, speech-language therapists, and dietitians. For children with limited oropharyngeal motor skills, effective interventions include structured oral-motor training programs and tailoring food texture to their abilities [30]. Tailored care strategies that evolve across developmental stages are crucial to reduce complications, support adequate growth, and improve overall well-being.

A detailed overview of the above mentioned ENT manifestations, including prevalence, pathophysiological mechanisms, clinical implications, and management strategies, is presented in Table 2.

Integrated, continuous, and individualized care is essential to optimize developmental trajectories and improve overall quality of life for children with Down syndrome [1].

## 6. Conclusions

Down syndrome is characterized by a broad range of otorhinolaryngologic conditions, including conductive hearing loss, recurrent otitis media, obstructive sleep apnea syndrome, and craniofacial dysmorphisms such as macroglossia and midfacial hypoplasia [4]. These structural and functional anomalies contribute to significant impairments in feeding, swallowing, and speech development [45]. Early recognition of these issues, through targeted screening and comprehensive diagnostic evaluations, is essential to prevent secondary developmental delays and to optimize therapeutic outcomes. Effective management relies on a multidisciplinary team, supporting the implementation of individualized care plans that address clinical needs while promoting overall health, communication, and quality of life [31].

## 7. Future Directions

Despite growing awareness of ENT-related complications in children with Down syndrome, current literature still presents several limitations. There is a lack of large-scale, longitudinal studies assessing the long-term outcomes of early audiological interventions, particularly in relation to language development and quality of life. Moreover, standardized protocols for ENT monitoring and multidisciplinary coordination remain inconsistently applied across clinical settings. Future research should aim to define evidence-based pathways that integrate audiologic, speech, and respiratory care, while also evaluating the cost-effectiveness and accessibility of these interventions. Prospective studies comparing the outcomes of different management strategies could provide clearer clinical insights. Ultimately, achieving early and accurate diagnosis of otolaryngological disorders, and ensuring timely, coordinated, and minimally invasive management, represents a critical goal. Such an approach not only enhances clinical outcomes but also promotes better developmental trajectories and overall quality of life.

## Figures and Tables

**Table 1:** Comparison of hearing aids and tympanostomy tubes for managing conductive hearing loss in children with Down syndrome, [22,34,35].

Aspect	Hearing Aids	Grommets (Timpanostomy Tubes)
Efficacy	Comparable efficacy to grommets for mild-to-moderate CHL	Effective for recurrent OME and AOM, especially with effusion
Invasiveness	Non-invasive	Invasive, require general anaesthesia
Risks	Minimal, skin irritation or device intolerance	Risks include otorrhea, tympanic membrane perforation, anaesthesia complications
Parental Perception	Preferred by many parents due to reduced emotional stress	Often associated with increased parental anxiety
Maintenance	Require regular monitoring and fitting	May require reinsertion or replacement; risk of extrusion
Indications	Persistent CHL without infection; non-surgical candidates	Recurrent AOM, OME > 3 months, failed conservative therapy
Contraindications	Significant hyperactivity, sensory intolerance	Craniofacial abnormalities or anaesthesia risks

**Table 2:** Overview of the most prevalent ENT disorders in children with Down syndrome.

ENT Condition	Estimated Prevalence	Key Causes	Clinical Consequences	Management	Reference
Conductive Hearing Loss	48.8%	OME, eustachian tube dysfunction, craniofacial anomalies	Language delay, articulation disorders	Hearing aids, monitoring	[16]
Sensorineural Hearing Loss	4.1%	Inner ear malformations, cochlear nerve dysfunction	Permanent hearing loss, speech impairment	Early diagnosis, cochlear implant (selected cases)	[16]
Otitis Media with Effusion	35.8%	URTI, adenoid hypertrophy, Immune deficiency	Fluctuating hearing, CHL	Tympanometry, PETs, medical therapy	[16]
Obstructive Sleep Apnea	45–76%	Macroglossia, midfacial hypoplasia, hypotonia, obesity	Cognitive decline, pulmonary hypertension	Polysomnography, adenotonsillectomy, CPAP	[5]
Dysphagia	>50%	Oromotor hypotonia, macroglossia, coordination issues	Aspiration risk, malnutrition	Feeding therapy, nutritional support	[15]
Language and Speech Delay	Nearly universal	Hearing loss, hypotonia, articulatory difficulty	Communication impairment, learning delay	Speech-language therapy, augmentative and alternative communication	[12]

## Data Availability

The data presented in this study are available in the cited references.
